# Supplementary material for: HIVprotI: an integrated web based platform for prediction and design of HIV proteins inhibitors
Source: J Cheminform. 2018 Mar 9;10:12. doi: 10.1186/s13321-018-0266-y (PMC5845081; doi:10.1186/s13321-018-0266-y)
Supplement: Supplementary file 2 — Additional file 2. Source code of HIVProtI web server. [file 13321_2018_266_MOESM2_ESM.zip › HIVprotI_Source-code/hivprotI/weka-3-6-6/doc/weka/classifiers/functions/LinearRegression.html]

LinearRegression


---


|  |  |  |
| --- | --- | --- |
| **Overview** \| **Package** \| **Class** \| **Tree** \| **Deprecated** \| **Index** \| **Help** \| **Weka's home** | |  |
| **PREV CLASS**   **NEXT CLASS** | **FRAMES**    **NO FRAMES**     **All Classes** |  |
| SUMMARY: NESTED \| FIELD \| CONSTR \| METHOD | DETAIL: FIELD \| CONSTR \| METHOD |  |


---


## weka.classifiers.functions Class LinearRegression

```
java.lang.Object
  weka.classifiers.Classifier
      weka.classifiers.functions.LinearRegression
```

**All Implemented Interfaces:**: java.io.Serializable, java.lang.Cloneable, CapabilitiesHandler, OptionHandler, RevisionHandler, WeightedInstancesHandler

---

``` public class LinearRegression extends Classifier implements OptionHandler, WeightedInstancesHandler ```

Class for using linear regression for prediction. Uses the Akaike criterion for model selection, and is able to deal with weighted instances.


Valid options are:

```
 -D
  Produce debugging output.
  (default no debugging output)
```

```
 -S <number of selection method>
  Set the attribute selection method to use. 1 = None, 2 = Greedy.
  (default 0 = M5' method)
```

```
 -C
  Do not try to eliminate colinear attributes.
```

```
 -R <double>
  Set ridge parameter (default 1.0e-8).
```

**Version:**
:   $Revision: 5523 $

**Author:**
:   Eibe Frank (eibe@cs.waikato.ac.nz), Len Trigg (trigg@cs.waikato.ac.nz)

**See Also:**: Serialized Form

---

| **Field Summary** | |
| --- | --- |
| `static int` | `SELECTION_GREEDY`             Attribute selection method: Greedy method |
| `static int` | `SELECTION_M5`             Attribute selection method: M5 method |
| `static int` | `SELECTION_NONE`             Attribute selection method: No attribute selection |
| `static Tag[]` | `TAGS_SELECTION`             Attribute selection methods |


**Constructor Summary**

`LinearRegression()`


| **Method Summary** | |
| --- | --- |
| `java.lang.String` | `attributeSelectionMethodTipText()`             Returns the tip text for this property |
| `void` | `buildClassifier(Instances data)`             Builds a regression model for the given data. |
| `double` | `classifyInstance(Instance instance)`             Classifies the given instance using the linear regression function. |
| `double[]` | `coefficients()`             Returns the coefficients for this linear model. |
| `java.lang.String` | `debugTipText()`             Returns the tip text for this property |
| `java.lang.String` | `eliminateColinearAttributesTipText()`             Returns the tip text for this property |
| `SelectedTag` | `getAttributeSelectionMethod()`             Gets the method used to select attributes for use in the linear regression. |
| `Capabilities` | `getCapabilities()`             Returns default capabilities of the classifier. |
| `boolean` | `getDebug()`             Controls whether debugging output will be printed |
| `boolean` | `getEliminateColinearAttributes()`             Get the value of EliminateColinearAttributes. |
| `java.lang.String[]` | `getOptions()`             Gets the current settings of the classifier. |
| `java.lang.String` | `getRevision()`             Returns the revision string. |
| `double` | `getRidge()`             Get the value of Ridge. |
| `java.lang.String` | `globalInfo()`             Returns a string describing this classifier |
| `java.util.Enumeration` | `listOptions()`             Returns an enumeration describing the available options. |
| `static void` | `main(java.lang.String[] argv)`             Generates a linear regression function predictor. |
| `int` | `numParameters()`             Get the number of coefficients used in the model |
| `java.lang.String` | `ridgeTipText()`             Returns the tip text for this property |
| `void` | `setAttributeSelectionMethod(SelectedTag method)`             Sets the method used to select attributes for use in the linear regression. |
| `void` | `setDebug(boolean debug)`             Controls whether debugging output will be printed |
| `void` | `setEliminateColinearAttributes(boolean newEliminateColinearAttributes)`             Set the value of EliminateColinearAttributes. |
| `void` | `setOptions(java.lang.String[] options)`             Parses a given list of options. |
| `void` | `setRidge(double newRidge)`             Set the value of Ridge. |
| `java.lang.String` | `toString()`             Outputs the linear regression model as a string. |
| `void` | `turnChecksOff()`             Turns off checks for missing values, etc. |
| `void` | `turnChecksOn()`             Turns on checks for missing values, etc. |

**Methods inherited from class weka.classifiers.Classifier**

`distributionForInstance, forName, makeCopies, makeCopy`

**Methods inherited from class java.lang.Object**

`equals, getClass, hashCode, notify, notifyAll, wait, wait, wait`

**Field Detail**

### SELECTION\_M5

```
public static final int SELECTION_M5
```

:   Attribute selection method: M5 method

    **See Also:**: Constant Field Values

---


### SELECTION\_NONE

```
public static final int SELECTION_NONE
```

:   Attribute selection method: No attribute selection

    **See Also:**: Constant Field Values

---


### SELECTION\_GREEDY

```
public static final int SELECTION_GREEDY
```

:   Attribute selection method: Greedy method

    **See Also:**: Constant Field Values

---


### TAGS\_SELECTION

```
public static final Tag[] TAGS_SELECTION
```

:   Attribute selection methods


**Constructor Detail**

### LinearRegression

```
public LinearRegression()
```


**Method Detail**

### turnChecksOff

```
public void turnChecksOff()
```

:   Turns off checks for missing values, etc. Use with caution.
    Also turns off scaling.

---


### turnChecksOn

```
public void turnChecksOn()
```

:   Turns on checks for missing values, etc. Also turns
    on scaling.

---


### globalInfo

```
public java.lang.String globalInfo()
```

:   Returns a string describing this classifier

    :   **Returns:**: a description of the classifier suitable for displaying in the explorer/experimenter gui

---


### getCapabilities

```
public Capabilities getCapabilities()
```

:   Returns default capabilities of the classifier.

    :   **Specified by:**: `getCapabilities` in interface `CapabilitiesHandler` **Overrides:**: `getCapabilities` in class `Classifier`
    :   **Returns:**: the capabilities of this classifier **See Also:**: `Capabilities`

---


### buildClassifier

```
public void buildClassifier(Instances data)
                     throws java.lang.Exception
```

:   Builds a regression model for the given data.

    :   **Specified by:**: `buildClassifier` in class `Classifier`
    :   **Parameters:**: `data` - the training data to be used for generating the linear regression function **Throws:**: `java.lang.Exception` - if the classifier could not be built successfully

---


### classifyInstance

```
public double classifyInstance(Instance instance)
                        throws java.lang.Exception
```

:   Classifies the given instance using the linear regression function.

    :   **Overrides:**: `classifyInstance` in class `Classifier`
    :   **Parameters:**: `instance` - the test instance **Returns:**: the classification **Throws:**: `java.lang.Exception` - if classification can't be done successfully

---


### toString

```
public java.lang.String toString()
```

:   Outputs the linear regression model as a string.

    :   **Overrides:**: `toString` in class `java.lang.Object`
    :   **Returns:**: the model as string

---


### listOptions

```
public java.util.Enumeration listOptions()
```

:   Returns an enumeration describing the available options.

    :   **Specified by:**: `listOptions` in interface `OptionHandler` **Overrides:**: `listOptions` in class `Classifier`
    :   **Returns:**: an enumeration of all the available options.

---


### setOptions

```
public void setOptions(java.lang.String[] options)
                throws java.lang.Exception
```

:   Parses a given list of options.


    Valid options are:

    ```
     -D
      Produce debugging output.
      (default no debugging output)
    ```

    ```
     -S <number of selection method>
      Set the attribute selection method to use. 1 = None, 2 = Greedy.
      (default 0 = M5' method)
    ```

    ```
     -C
      Do not try to eliminate colinear attributes.
    ```

    ```
     -R <double>
      Set ridge parameter (default 1.0e-8).
    ```

    :   **Specified by:**: `setOptions` in interface `OptionHandler` **Overrides:**: `setOptions` in class `Classifier`
    :   **Parameters:**: `options` - the list of options as an array of strings **Throws:**: `java.lang.Exception` - if an option is not supported

---


### coefficients

```
public double[] coefficients()
```

:   Returns the coefficients for this linear model.

    :   **Returns:**: the coefficients for this linear model

---


### getOptions

```
public java.lang.String[] getOptions()
```

:   Gets the current settings of the classifier.

    :   **Specified by:**: `getOptions` in interface `OptionHandler` **Overrides:**: `getOptions` in class `Classifier`
    :   **Returns:**: an array of strings suitable for passing to setOptions

---


### ridgeTipText

```
public java.lang.String ridgeTipText()
```

:   Returns the tip text for this property

    :   **Returns:**: tip text for this property suitable for displaying in the explorer/experimenter gui

---


### getRidge

```
public double getRidge()
```

:   Get the value of Ridge.

    :   **Returns:**: Value of Ridge.

---


### setRidge

```
public void setRidge(double newRidge)
```

:   Set the value of Ridge.

    :   **Parameters:**: `newRidge` - Value to assign to Ridge.

---


### eliminateColinearAttributesTipText

```
public java.lang.String eliminateColinearAttributesTipText()
```

:   Returns the tip text for this property

    :   **Returns:**: tip text for this property suitable for displaying in the explorer/experimenter gui

---


### getEliminateColinearAttributes

```
public boolean getEliminateColinearAttributes()
```

:   Get the value of EliminateColinearAttributes.

    :   **Returns:**: Value of EliminateColinearAttributes.

---


### setEliminateColinearAttributes

```
public void setEliminateColinearAttributes(boolean newEliminateColinearAttributes)
```

:   Set the value of EliminateColinearAttributes.

    :   **Parameters:**: `newEliminateColinearAttributes` - Value to assign to EliminateColinearAttributes.

---


### numParameters

```
public int numParameters()
```

:   Get the number of coefficients used in the model

    :   **Returns:**: the number of coefficients

---


### attributeSelectionMethodTipText

```
public java.lang.String attributeSelectionMethodTipText()
```

:   Returns the tip text for this property

    :   **Returns:**: tip text for this property suitable for displaying in the explorer/experimenter gui

---


### setAttributeSelectionMethod

```
public void setAttributeSelectionMethod(SelectedTag method)
```

:   Sets the method used to select attributes for use in the
    linear regression.

    :   **Parameters:**: `method` - the attribute selection method to use.

---


### getAttributeSelectionMethod

```
public SelectedTag getAttributeSelectionMethod()
```

:   Gets the method used to select attributes for use in the
    linear regression.

    :   **Returns:**: the method to use.

---


### debugTipText

```
public java.lang.String debugTipText()
```

:   Returns the tip text for this property

    :   **Overrides:**: `debugTipText` in class `Classifier`
    :   **Returns:**: tip text for this property suitable for displaying in the explorer/experimenter gui

---


### setDebug

```
public void setDebug(boolean debug)
```

:   Controls whether debugging output will be printed

    :   **Overrides:**: `setDebug` in class `Classifier`
    :   **Parameters:**: `debug` - true if debugging output should be printed

---


### getDebug

```
public boolean getDebug()
```

:   Controls whether debugging output will be printed

    :   **Overrides:**: `getDebug` in class `Classifier`
    :   **Returns:**: true if debugging output is printed

---


### getRevision

```
public java.lang.String getRevision()
```

:   Returns the revision string.

    :   **Specified by:**: `getRevision` in interface `RevisionHandler` **Overrides:**: `getRevision` in class `Classifier`
    :   **Returns:**: the revision

---


### main

```
public static void main(java.lang.String[] argv)
```

:   Generates a linear regression function predictor.

    :   **Parameters:**: `argv` - the options


---


|  |  |  |
| --- | --- | --- |
| **Overview** \| **Package** \| **Class** \| **Tree** \| **Deprecated** \| **Index** \| **Help** \| **Weka's home** | |  |
| **PREV CLASS**   **NEXT CLASS** | **FRAMES**    **NO FRAMES**     **All Classes** |  |
| SUMMARY: NESTED \| FIELD \| CONSTR \| METHOD | DETAIL: FIELD \| CONSTR \| METHOD |  |


---
